# Prediction of Mechanical Properties of Nano-Clay-Based Biopolymeric Composites

**DOI:** 10.3390/nano14171403

**Published:** 2024-08-28

**Authors:** Rodica Cristina Voicu, Mihai Gologanu, Catalin Tibeica, Mercedes Santiago-Calvo, María Asensio, Esteban Cañibano, Oana Nedelcu, Titus Sandu

**Affiliations:** 1 National Institute for Research and Development in Microtechnologies—IMT Bucharest, 126A, Erou Iancu Nicolae Street, 077190 Voluntari, Ilfov, Romaniacatalin.tibeica@imt.ro (C.T.);; 2 Foundation for Research and Development in Transport and Energy—FUNDACIÓN CIDAUT, Parque Tecnológico de Boecillo, 47051 Boecillo, Spain; mersan@cidaut.es (M.S.-C.);

**Keywords:** nanocomposite, nano-clay, polymer, mechanical properties, modeling, simulation

## Abstract

An understanding of the mechanical behavior of polymeric materials is crucial for making advancements in the applications and efficiency of nanocomposites, and encompasses their service life, load resistance, and overall reliability. The present study focused on the prediction of the mechanical behavior of biopolymeric nanocomposites with nano-clays as the nanoadditives, using a new modeling and simulation method based on Comsol Multiphysics software 6.1. This modeling considered the complex case of flake-shaped nano-clay additives that could form aggregates along the polymeric matrix, varying the nanoadditive thickness, and consequently affecting the resulting mechanical properties of the polymeric nanocomposite. The polymeric matrix investigated was biopolyamide 11 (BIOPA11). Several BIOPA11 samples reinforced with three different contents of nano-clays (0, 3, and 10 wt%), and with three different nano-clay dispersion grades (employing three different extrusion screw configurations) were obtained by the compounding extrusion process. The mechanical behavior of these samples was studied by the experimental tensile test. The experimental results indicate an enhancement of Young’s modulus as the nano-clay content was increased from 0 to 10 wt% for the same dispersion grades. In addition, the Young’s modulus value increased when the dispersion rate of the nano-clays was improved, showing the highest increase of around 93% for the nanocomposite with 10 wt% nano-clay. A comparison of the modeled mechanical properties and the experimental measurements values was performed to validate the modeling results. The simulated results fit well with the experimental values of Young’s modulus.

## 1. Introduction

Polymers are extensively employed in a wide range of applications, owing to their inherent multifunctional properties, cost-effectiveness, and ease of fabrication. To expand their applications and enhance their functionality, durability, reliability, and cost-effectiveness, polymers are reinforced with fillers to improve their mechanical properties, specifically their stiffness and strength [1,2]. Over the past decade, various types of nanoadditives/nanofillers, including nano-clay, carbon nanotubes (CNTs), and graphene, have attracted substantial interest in researchers’ works. One of the nanoadditive of interest is mineral clay consisting of layered silicates, which form intricate clay crystallites by the stacking of layered structural units. These materials possess distinctive characteristics, shaped by the composition of their individual units, which include octahedral and/or tetrahedral sheets. Specifically, the tetrahedral sheets in nano-clays consist of interconnected silicon–oxygen tetrahedra, where each tetrahedron shares three corners with adjacent tetrahedra. Furthermore, the fourth vertex of each tetrahedral sheet forms a covalent bond with a neighboring octahedral sheet [3]. Due to the synergic effects of their constituent characteristics, clay-based polymer nanocomposites demonstrate superior mechanical properties, diffusion barriers, fire retardancy, and ultra-violet (UV) resistance compared to their micro/macro counterparts and pure polymeric resins. Nanofillers, characterized by having at least one dimension in the nanometer range, exhibit morphologies that vary from isotropic to highly anisotropic. The uniform dispersion of these nanofillers, whether isotropic or anisotropic, results in a significantly large interfacial area between the nanoadditives and the polymer matrix [1,2]. This extensive interfacial area imparts superior properties to the base polymer. Nanoadditives can improve the mechanical properties, including strength, stiffness, and toughness, as well as enhance the thermal and electrical conductivities of polymers. Additionally, they can introduce specific functionalities for targeted applications [1,2,3,4,5]. It is remarkable that polymer nanocomposites need very small amounts of clay (below 10 wt% clay) to exhibit their unique properties, due to their nanoscale structural behavior [6,7]. Moreover, the interaction between the polymer chains and the clay layers creates strong interfacial bonding, which helps to distribute stress more evenly throughout the material, leading to improved overall performance. Several types of interactions can occur: (1) physical adsorption involves polymer chains adsorbing onto clay surfaces through van der Waals forces and hydrogen bonding; (2) ionic interactions occur when the exchangeable cations in nano-clays bond with the polar groups in polymers; and (3) covalent bonding, achieved through the chemical modifiers of the clays, forms strong interfacial bonds between the clay and polymer chains. Therefore, these chemical interactions make nano-clays an ideal additive for producing high-performance polymer nanocomposites [8,9]. A critical challenge for producing polymer–clay nanocomposites is achieving a uniform dispersion of the clay within the polymer matrix to maximize performance. Recent research has focused on understanding the techniques that facilitate clay dispersion in polymer matrices. It has been noted that clay dispersion is influenced by the processing conditions, melt rheology, the clay’s structure, and the structure of the clay modifier [1,2,3,4,5,6,7,8,9,10].

The enhancement of both the mechanical and functional properties of clay-based polymer nanocomposites positions them as promising candidate for a wide range of applications, including in the packaging, transportation, automotive, aerospace, electronics, and medical sectors [10]. Primarily, petro-based polymer nanocomposites are used in these industries, but there is increasing interest in using bio-based matrices to replace polymers with fossil fuel origins, due to their sustainable and environmentally friendly properties. Thus, biopolymer nanocomposites are an exciting area of research and development, combining biopolymers with nanoadditives to create new, sustainable, and eco-friendly alternatives for various industrial applications [11,12].

For the continued advancement of clay-based polymer nanocomposites, including those with bio-based matrices, a thorough understanding of their mechanical behavior and properties is essential. Most studies in the literature have focused on the processing and/or experimental characterizations of clay-based polymer nanocomposites [10], with less emphasis on the predictive tools for determining the mechanical properties of this emerging class of materials, which is crucial for realizing their potential in industrial applications.

The theoretical investigations into predicting the mechanical behavior of nano-clay-reinforced polymers can be categorized into three main approaches: atomistic modeling, continuum modeling (both analytical and numerical), and multi-scale methods [10]. The numerical continuum modeling of a selected representative volume element (RVE) is typically performed using finite element methods (FEMs), which can account for a material’s nonlinearity. Nano-clay-based polymer nanocomposites are inherently a 3D medium due to the random orientations of their inclusions, necessitating the consideration of diverse particle shapes to accurately predict their mechanical properties. However, the complexity and time-consuming nature of these simulations limit the applicability of this method for RVEs with various inclusion shapes. Therefore, most FE studies used disk-shaped or square-plate particles for nano-clays, with slender rectangles representing the high aspect ratios of the nano-clay platelets often used in 2D analysis. Significant complexities arise in the FE modeling and analysis of a material region containing high-aspect-ratio particles. Additionally, modeling the interphase between nano-clays and polymers with numerical continuum modeling presents another challenge [10,11,12,13,14,15,16].

The literature reveals a noticeable gap in the development of proper modeling techniques for characterizing the mechanical properties of nano-clay-based polymer nanocomposites, which consider all the involved parameters [10]. Therefore, this field remains an ongoing area of research, requiring a deeper understanding of the underlying physical phenomena. 

Thus, this paper focuses on the prediction of the mechanical properties of nano-clay-based polymeric nanocomposites using a numerical homogenization method based on a finite element model of a periodic RVE. By varying the aspect ratio of the RVE at a constant nanoadditives concentration, we were able to study the effect of neighboring inclusions and their agglomeration along particular preferred directions. All the simulations were run using COMSOL Multiphysics. The polymeric matrix investigated was biopolyamide 11 (BIOPA11). At the end, a comparison between the modeled mechanical properties and the experimental measurements values was performed, and the prediction of Young’s modulus for the different nanoadditives concentrations in the matrix was successful.

## 2. Materials and Methods

This section outlines the materials, preparation techniques, and the modeling methods employed for the nanocomposites. 

### 2.1. Materials

**Figure 1:**
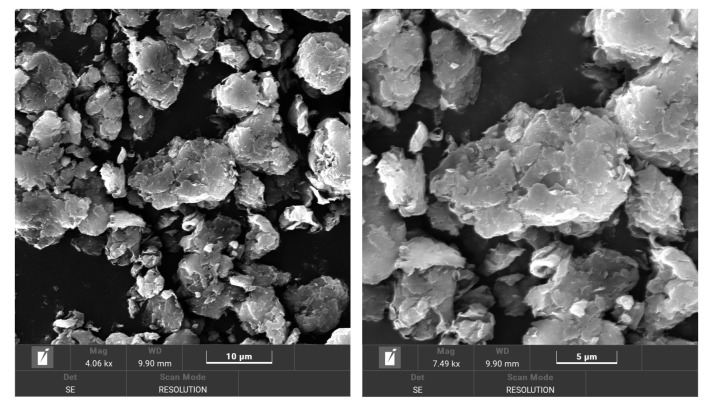
SEM micrographs of Cloisite 20A nano-clay particles.

Rilsan KNO resin from Arkema is a biopolyamide 11 (BIOPA11), employed as a 100% bio-based polymer. This biopolyamide grade has a density of 1.03 g/cm^3^ (ISO 1183-1:2019 [17]) and a melting temperature of 189 °C (ISO 11357-1:2023 [18]). Also, its tensile modulus and yield strain are 1250 MPa and 37 MPa, respectively. Cloisite 20A nano-clay from Southern Clay Products, Inc (Gonzales, LA, USA), supplied by BYK-Chemie GmbH (Wesel, Germany), was used as the nanofiller to obtain bionanocomposites based on BIOPA11. This nano-clay is an organophilic montmorillonite modified with dimethyl dihydrogenated tallow quaternary ammonium chloride. This quaternary ammonium salt modifier replaces the silanol groups of natural organoclay, making it more compatible with a polymer matrix. Cloisite 20A nano-clays are layered silicates with lateral sizes in the micrometric dimension (particle size: D10 < 2 µm, D50 < 10 µm, D90 < 13 µm), and the thickness of the individual layers in the nanoscale (lamellar spacing: 2.7 nm, measured by X-Ray Diffraction d-Spacing, d001). The morphology of the Cloisite20A can be observed from the SEM micrographs displayed in Figure 1.

### 2.2. Nanocomposite Preparation

The polymeric nanocomposites were prepared by compounding extrusion using a Leistritz 27 GL L/D 36 co-rotating twin-screw extruder. To study the effect of the amount of reinforcements on the neat BIOPA11 polymeric matrix, three ratios of nano-clays were selected: 0, 3, and 10 wt%. These nano-clay contents were selected by considering that previous studies with amounts below 10 wt% have been able to improve the properties of the base polymer [6,7]. In addition, three different screw configurations for the extrusion process were evaluated to increase the dispersion capacity of the nano-clays (Table 1). In particular, the three different screw configurations generated different levels of shear stress, as follows: Screw 1: medium shear stress; Screw 2: high shear stress; and Screw 3: very high shear stress. The processing parameters employed for all the nanocomposites were as follows: the temperature profile of the barrel ranged from 180 to 240 °C (the temperature of the extruder barrel elements (°C) were 180, 180, 190, 200, 210, 220, 230, 240, 240 (die)), the manufacturing speed was 5 kg/h, and the screw rotation speed was 150 rpm.

### 2.3. Tensile Properties of Nanocomposites

**Figure 2:**
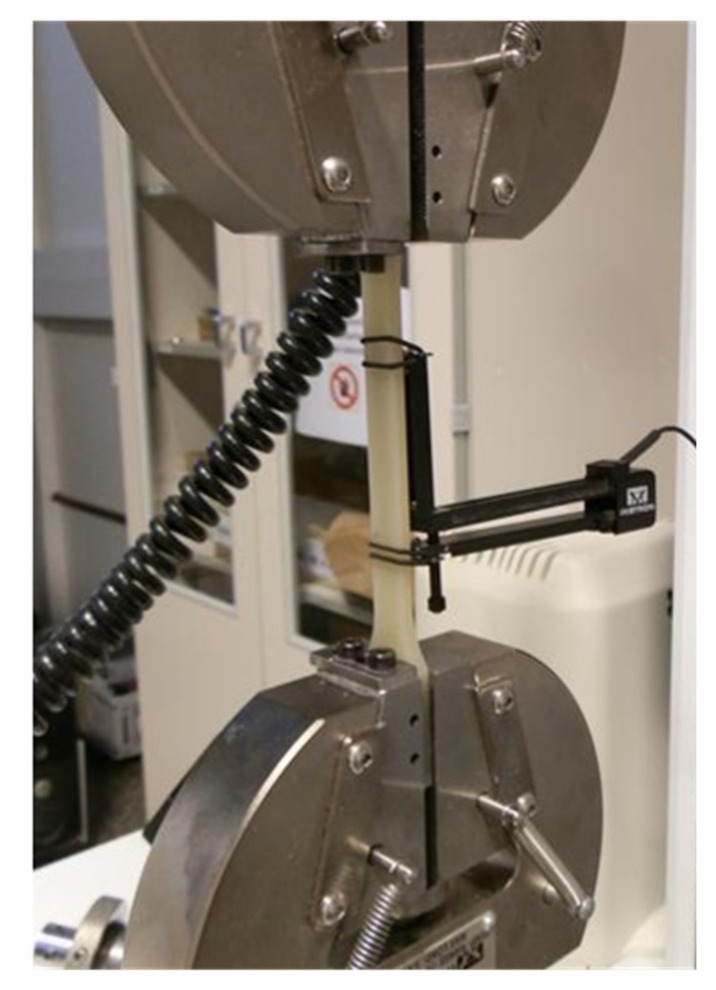
Example of tensile testing of type 1A specimen.

The tensile properties of the nanocomposites produced with the different screw configurations and formulations were characterized using an Instron Model 5500R60025 at a speed of 1 mm/min and 50 mm/min, following the general principles outlined in ISO 527-1:2019 [19] (Figure 2). The tensile test specimens, with type 1A dimensions, were produced using a Krauss Maffei KM 200 injection molding machine. The temperature profile of the injection cylinders was set to 250 °C, and the mold temperature was maintained at 60 °C. In this tensile test, Young’s modulus, defined as the slope of the stress–strain curve, was measured and expressed in megapascals (MPa).

### 2.4. Modeling of the Nancomposite Material

Numerical simulations were performed with COMSOL Multiphysics software 6.1, with the aim of determining Young’s modulus for the all nanocomposites with 3 wt% and 10 wt% nano-clay concentrations in the polymer’s matrices.

A simplified micromechanical model of a particulate composite was used to estimate the mechanical properties of the nanocomposites. A representative volume element (RVE) based on a predetermined particle spacing was assumed to represent the microstructure of the composite. The homogenized elastic properties of the composite material were computed based on the individual properties of the particles and the matrix.

**Figure 3:**
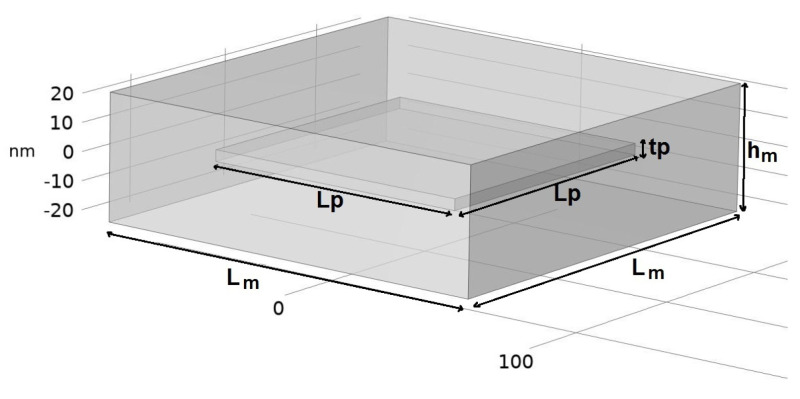
The geometry of the unit cell, consisting of a nano-flake particle embedded in the polymer matrix.

The composite was assumed to be made of a periodic microstructure identified as a primitive parallelepiped structure. A unit parallelepiped RVE, having a parallelepiped particle (the flake of nanoadditive) embedded in the center of the matrix, is shown in Figure 3. The notations used for the lengths and thicknesses are presented in Table 2.

Although the thickness of a single flake is about 1 nm, we considered the thickness as a variable in our model, due to the possible agglomeration of several flakes into a cluster.

The properties of the materials used for the simulations were chosen from the literature and also from the measurements performed. The initial Young’s modulus for BIOPA11 was set at 1.192 GPa from the measurements, and the Poisson ratio was set at 0.3, determined from the literature as a typical value for polymers [20]. For the nano-clay flakes, we found a wide range of values for its Young’s modulus in the literature, but we fixed it at an average value of 150 GPa and a Poisson ratio of 0.22 [10]. Different obstacles to defining a specific value for nano-clay’s Young’s modulus can appear [10]. It is well-known that performing direct tests on a single clay platelet is impossible. Also, there are always differences between clays’ nano-structures, and there is uncertainty in the data acquired from indirect measurements. High costs are incurred when performing nanoscale experiments, and variations in the experimental measurements obtained can appear from one sample to another.

The micromechanical analysis of the particles in a bulk matrix can be performed using the Cell Periodicity node option in the Solid Mechanics interface in Comsol. Using this function, the elasticity matrix of the homogenized material can be computed for a given particle and matrix properties.

We have to mention that the Young’s modulus of the polymer matrix and of the flakes strongly influence the nanocomposite’s simulated Young’s modulus.

In order to define the dimensions of the unit cell, we had to fix a length for the polymer matrix and a thickness for the flakes (we supposed the flakes are stacked to form a thickness together, tp nm). We varied them, keeping the length of the flakes at 100 nm × 100 nm. After fixing the geometry of the unit cell, we performed new simulations in order to obtain the Young’s modulus (elasticity matrix) results when the concentration of flakes in the polymer matrix was varied. For the initial stage, the mass concentration was fixed at 3 wt%, as provided by the experimental measurements.

For the variation in concentration, C%, and $L_{m}$ we used (1)

(1) $$C=\frac{100\cdot 100\cdot tp}{L_{m}\cdot L_{m}\cdot h_{m}}$$

We considered the materials to be orthotropic and we simulated the elasticity matrix in the Standard (11, 22, 33, 12, 23, 13) components; after that, we computed the inverse matrix in order to identify the $E_{x}$, $E_{y}$, and $E_{z}$ components of the elasticity matrix.

The elasticity matrix, *D*, obtained from the simulations, has the following structure (2) [21]:

(2) $$D=\left[\begin{array}{cc} \begin{array}{ccc} D_{11} & D_{12} & D_{13}\\ D_{12} & D_{22} & D_{23}\\ D_{13} & D_{23} & D_{33} \end{array} & \begin{array}{ccc} 0 & \;0\; & \;0\\ 0 & \;0 & \;0\\ 0 & \;0 & \;0 \end{array}\\ \begin{array}{ccc} 0 & \;0 & \;0\\ 0 & \;0 & \;0\\ 0 & \;0 & \;0 \end{array} & \begin{array}{ccc} D_{44} & \;0 & 0\\ 0 & D_{55} & 0\\ 0 & 0 & D_{66} \end{array} \end{array}\right]\;$$

where

$$D_{11}=\frac{E_{x}^{2}(E_{z}v_{yz}^{2}-E_{y})}{D_{denom}},\;D_{12}=\frac{E_{x}E_{y}(E_{z}v_{yz}v_{xz}+E_{y}v_{xy})}{D_{denom}}$$

$$D_{13}=\frac{E_{x}E_{y}E_{z}(v_{xy}v_{yz}+v_{xz})}{D_{denom}},\;D_{22}=\frac{E_{y}^{2}(E_{z}v_{xz}^{2}-E_{x})}{D_{denom}}$$

$$D_{23}=\frac{E_{y}E_{z}({E_{y}v}_{xy}v_{xz}+E_{x}v_{yz})}{D_{denom}},\;D_{33}=\frac{E_{y}E_{z}(E_{y}v_{xy}^{2}-E_{x})}{D_{denom}}$$

$$D_{denom}=E_{y}E_{z}v_{xz}^{2}-E_{x}E_{y}+2v_{xy}v_{yz}v_{xz}E_{y}E_{z}+E_{x}E_{z}v_{yz}^{2}+E_{y}^{2}v_{xy}^{2}.$$

The compliance matrix, the inverse matrix of *D*, *D*^−1^, was also computed, and we used it to extract the Young’s modulus $E_{x}$, $E_{y}$, and $E_{z}$ components (3).

(3) $$D=\left[\begin{array}{cc} \begin{array}{ccc} \frac{1}{E_{x}} & -\frac{v_{yx}}{E_{y}} & -\frac{v_{zx}}{E_{z}}\\ -\frac{v_{xy}}{E_{x}} & \frac{1}{E_{y}} & -\frac{v_{zy}}{E_{z}}\\ -\frac{v_{xz}}{E_{x}} & -\frac{v_{yz}}{E_{y}} & \frac{1}{E_{z}} \end{array} & \begin{array}{ccc} 0 & \;0\; & \;0\\ 0 & \;0 & \;0\\ 0 & \;0 & \;0 \end{array}\\ \begin{array}{ccc} 0 & \;0 & \;0\\ 0 & \;0 & \;0\\ 0 & \;0 & \;0 \end{array} & \begin{array}{ccc} \frac{1}{D_{44}} & \;0 & 0\\ 0 & {\frac{1}{D}}_{55} & 0\\ 0 & 0 & {\frac{1}{D}}_{66} \end{array} \end{array}\right]$$

**Figure 4:**
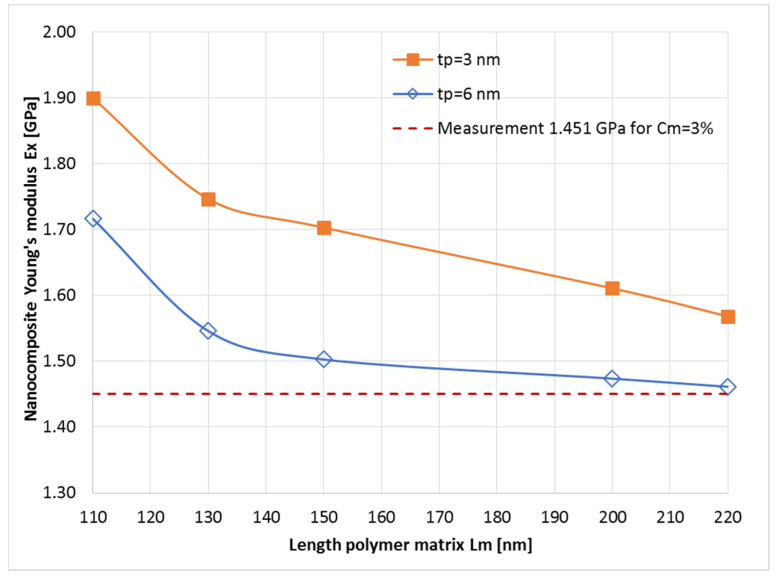
The nanocomposite Young’s modulus ($E_{x}$ component) when the length of the polymer matrix (BioPA11, E = 1.192 GPa) and the flake thickness are both varied, for a fixed mass concentration $C_{m}$ = 3 wt%.

Figure 4 presents the Young’s modulus ($E_{x}$ component) results for the modeling of each nanocomposite, for a mass concentration of 3 wt%, and their comparison with the measurements. From these simulation results, we obtained the optimized geometry of the unit cell, which was further used to simulate Young’s modulus for the nanocomposites as a function of flakes concentration, such that, for the polymer BIOPA11 with E = 1.192 GPa (0 wt%), we used a geometry of the unit cell with $L_{m}$ = 220 nm, *tp* = 6 nm, and Equation (1).

## 3. Results

This section presents the experimental measurements of the nanocomposite mechanical properties and the modeling and simulation results.

### 3.1. Young’s Modulus Measurements

Young’s modulus, also known as the modulus of elasticity, quantifies a material’s ability to resist deformation under longitudinal tensile or compressive stress. Table 3 presents the Young’s modulus results for the bionanocomposites produced with different amounts of Cloisite 20A nano-clays and with different extrusion screw configurations. Moreover, Table 3 shows the percentage increment in Young’s modulus for the bionanocomposites with 3 wt% and 10 wt% compared to the reference sample, without nanoadditives and the same screw configurations.

As the data collected in Table 3 show, when the amount of particles increases from 0 to 10 wt%, the Young’s modulus value also increases with the same screw configuration [22]. The differences become more significant between the reference sample (the neat polymer without nanoadditives) and the highest nanoadditive concentrations (10 wt%). In addition, the Young’s modulus value improves with a rise in the shear stress by means of the various screw configurations during the compounding process. The latter effect is related to the better dispersion rate of the nanoadditive in the biocomposite, thanks to the design of the extrusion screw [23]. Therefore, the enhancement of the Young’s modulus values is simultaneously due to the screw configuration and the nanoadditive content. The bionanocomposite sample (Screw 3, BioPA11_10 wt% nano-clays) extruded with 10 wt% Cloisite 20A nano-clays and with the Screw 3 configuration is the one that achieves the highest increase in Young’s modulus (around 93%) compared to the reference sample without additives. This is due to its high nano-clay content and the very high shear stress produced by the Screw 3 configuration, resulting in the improved dispersion of the nanoparticles. Moreover, the effective dispersion of the nano-clays within the biopolyamide matrix facilitates the intercalation and exfoliation of these nanoadditives, resulting in enhanced mechanical properties [24].

### 3.2. Modeling Results

**Figure 5:**
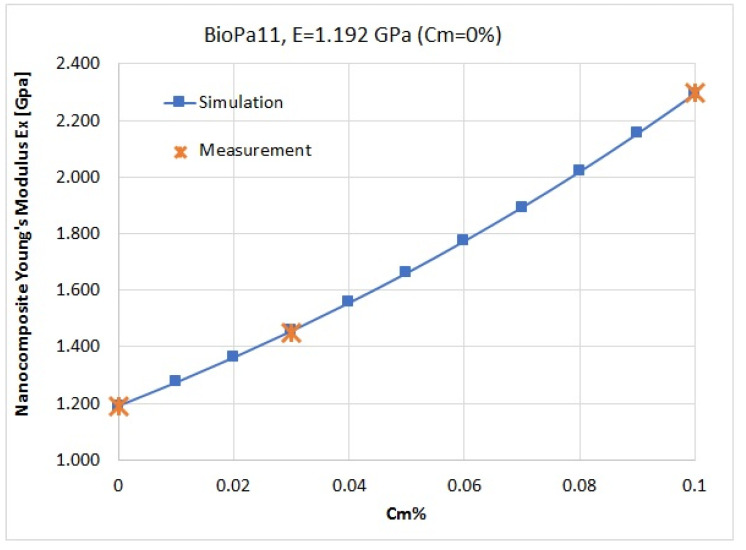
The nanocomposite’s Young’s modulus ($E_{x}$ component) when the flakes’ mass concentration is varied for BioPA11.

From the simulation results we obtained the optimized geometry of the unit cell, which was further used to simulate Young’s modulus for the nanocomposites as a function of the nano-clay concentration. The simulated results fit well with the measurements for both nanocomposites (for Screw 3 configuration). Then, the results for different concentrations in the polymer matrix were extended up to 10 wt %, giving us the opportunity to estimate the mechanical behavior of the nanocomposites for a larger range in the mass concentration of the nanoadditives, and to observe the behavior of the nanomaterials (Figure 5).

**Figure 6:**
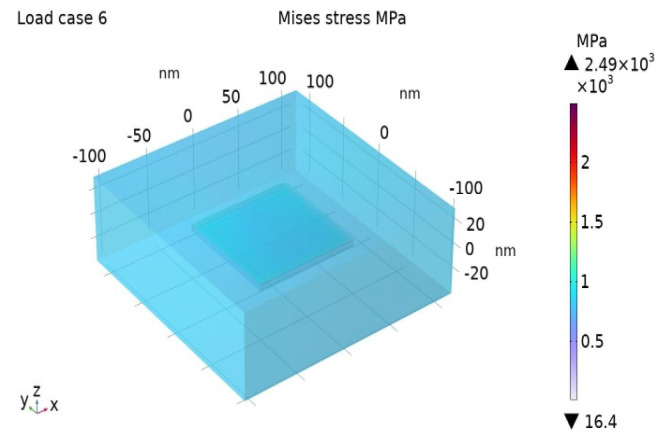
Misses stress for BioPA11 nanocomposite with $C_{m}$ = 3%.

**Figure 7:**
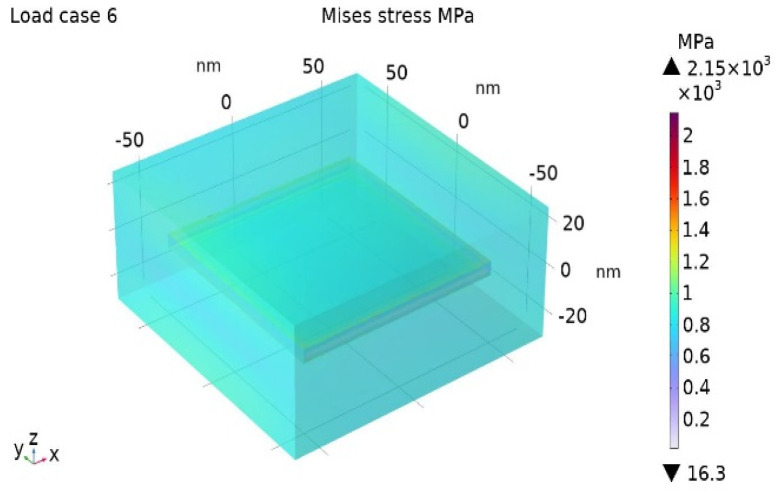
Misses stress for BioPA11 nanocomposite with $C_{m}$ = 10%.

The Misses stress distribution maps for the BIOPA11 nanocomposites with 3 wt% and 10 wt% nano-clays are shown in Figure 6 and Figure 7.

## 4. Discussion and Conclusions

Numerical continuum modeling can effectively estimate Young’s modulus for nano-clay-based biopolymer nanocomposites, providing accurate predictions of the mechanical properties of organoclay nanocomposites in some cases. However, critical issues, such as the interaction between nano-clays and polymers, as well as the formation of local aggregates, have been overlooked. In this paper, we tried to take into account this kind of aggregation by also varying the thicknesses of the nano-clay flakes. BIOPA11 samples reinforced with nano-clays (0 wt%, 3 wt%, and 10 wt%) were prepared by the compounding extrusion process, and three dispersion grades were employed using different extrusion screw configurations. The tensile mechanical properties of these samples were measured. The experimental results indicate that Young’s modulus increased as the nano-clay content was raised from 0 to 10 wt% for the same dispersion grade. Moreover, enhanced dispersion of the nano-clays led to a significant increase in Young’s modulus, with around an 93% improvement observed for the nanocomposite containing 10 wt% nano-clays. Numerical simulations were performed by using COMSOL Multiphysics software 6.1 to predict the mechanical properties of the nano-clay nanocomposites based on the BIOPA11 polymer matrix. The simulated results fitted well with the experimentally obtained values for concentrations of 0 wt%, 3 wt%, and 10 wt%, for the highest nano-clays dispersion. Based on these fitted values, the estimation and prediction of the Young’s modulus values were obtained for different concentrations between 1–10 wt%.

Further development of the modeling and simulation methods will include the homogenized viscoelastic property of the composite material to be computed, based on the individual properties of the particles and the polymer matrix. Transient analyses of the shear and normal loading of the composite microstructure will yield the viscoelastic response of the composite, which will be used to determine the homogenized viscoelastic parameters using curve-fitting optimization.

## Figures and Tables

**Table 1:** Formulations of nanocomposites used in compounding trials, combining different screws designs and amounts of additives.

Screw Configuration	Formulations of Nanocomposites		
Screw 1	BioPA11_0 wt% Nano-clays	BioPA11_3 wt% Nano-clays	BioPA11_10 wt% Nano-clays
Screw 2	BioPA11_0 wt% Nano-clays	BioPA11_3 wt% Nano-clays	BioPA11_10 wt% Nano-clays
Screw 3	BioPA11_0 wt% Nano-clays	BioPA11_3 wt% Nano-clays	BioPA11_10 wt% Nano-clays

**Table 2:** Notations for the representative volume element (RVE).

Geometry Dimension	Abbreviation	Unit [nm]
Length of the polymer matrix volume element	$L_{m}$	Varied between 110 nm and 220 nm
Thickness of the polymer matrix volume element	$h_{m}$	Varied as function of mass concentration, Cm%, and $L_{m}$
Length of the flake	$L_{p}$	100 nm × 100 nm
Thickness of the flake	tp	Varied between 1 nm and 9 nm

**Table 3:** Young’s modulus values for bionanocomposites obtained with different contents of nanoadditives and with different extrusion screw configurations.

	BioPA11_0 wt% Nano-Clays	BioPA11_3 wt% Nano-Clays		BioPA11_10 wt% Nano-Clays	
Screw Configur.	Young’s Modulus (MPa)	Young’s Modulus (MPa)	Increm. (%) *	Young’s Modulus (MPa)	Increm. (%) *
Screw 1	1219 ± 22	1344 ± 8	10.3	2023 ± 19	66.1
Screw 2	1205 ± 17	1435 ± 18	19.1	2090 ± 22	73.4
Screw 3	1192 ± 19	1451 ± 15	21.7	2298 ± 56	92.8

* Increm. (%) = the percentage increase in the Young’s modulus value when incorporating the nanoadditives compared to the neat polymer for the same screw configuration.

## Data Availability

The data are presented as graphs and tables in the manuscript.
